# Prognostic factors in HIV-positive patients with non-Hodgkin lymphoma: a Peruvian experience

**DOI:** 10.1186/s13027-018-0200-y

**Published:** 2018-07-31

**Authors:** Luis Ernesto Cuellar, Andrea Anampa-Guzmán, Alexis Manuel Holguín, Juan Velarde, Diana Portillo-Alvarez, Marco Antonio Zuñiga-Ninaquispe, Esther Rosa Luna-Reyes, Jule Vásquez, Joanne Marie Jeter, Karen Marie Winkfield

**Affiliations:** 1 Instituto Nacional de Enfermedades Neoplasicas, Lima, Peru; 2 Facultad de Medicina Humana, Universidad Nacional Mayor de San Marcos, Lima, Peru; 3 Divisions of Human Genetics and Medical Oncology, The Ohio State University, Columbus, OH USA; 4 Department of Radiation Oncology, Wake Forest Baptist Medical Center, Winston Salem, NC USA

## Abstract

**Background:**

Non-Hodgkin lymphoma (NHL) is the most common cancer in people with HIV. Although 95% of HIV patients are in developing countries like Peru, the majority of these studies have been conducted in developed countries. In this study we aim to evaluate prognostic factors associated with outcomes in HIV positive patients undergoing systemic therapy for treatment of NHL.

**Methods:**

This retrospective study includes patients with NHL seen in the Instituto Nacional de Enfermedades Neoplasicas (INEN) between 2004 to 2014. Patients were divided into two groups: antiretroviral therapy (ART) -naïve (*n* = 34) and those previously treated, ART-exposed (*n* = 13), at the time of diagnosis. All patients received chemotherapy and ART. The medical records were reviewed. Data were analyzed using t-test and chi-square test. Survival curves were estimated by the Kaplan-Meier method and comparison was done by log-rank test. Multivariate analysis for overall survival (OS) was performed with the Cox proportional hazard regression model.

**Results:**

All ART-exposed patients were from the capital city (*p* = 0.039); they had significantly lower hemoglobin levels compared to ART-naïve patients (*p* = 0.026). The median OS was 47.7 months with a 5-yr OS of 36.1%. The median OS for ART naïve patients was significantly higher than that for ART-exposed patients (57.05 and 21.09 months, respectively; *p* = 0.018). Advanced stage and low serum albumin were associated with lower OS in both groups. Age > 60 was associated with worse outcomes in the ART-naïve cohort.

**Conclusions:**

Advanced stage, low serum albumin and previous ART treatment were the primary prognostic factors associated with poorer outcomes in patients with NHL and HIV infection. In ART-naïve patients, age > 60 was associated with worse outcomes but in this cohort, older patients still had better overall outcomes than ART-exposed patients.

## Background

Non-Hodgkin lymphoma (NHL) is the most common cancer in patients with human immunodeficiency virus (HIV) and is one of the leading causes of death in this population [1]. NHL is an AIDS-defining disease in people infected with HIV-1. NHL incidence tends to be less affected by the use of antiretroviral therapy (ART) [2]. While the pandemic of HIV infection is still most pronounced in Sub-Saharan Africa, in 2016, 2 million people in Latin America and the Caribbean were living with HIV. Peru was one of the first countries in Latin America to offer prophylaxis from vertical transmission in pregnant woman. As of 2010, there were 72,000 people living with HIV in Peru, a prevalence of 0.3% [3]. That same year, there were 26,566 cases of AIDS, and NHL was noted to be the fifth most prevalent cancer [4].

Several studies have compared the evolution of NHL in the pre-ART and post-ART eras [5, 6]. A small number of studies have compared survival in patients starting ART when they are diagnosed with NHL (ART-naïve patients) and those who had previously received ART treatment at the time of their NHL diagnosis [7]. However, although 95% of HIV patients are in developing countries like Peru, the majority of these studies have been conducted in developed countries [8]. In an attempt to improve the limitations of previous studies, we studied the overall survival (OS) and prognostic factors in HIV-positive patients with systemic NHL in the post-ART era at the Intistituto Nacional de Enfermedades Neoplásicas (INEN) in Peru.

## Methods

This retrospective study included HIV-positive patients treated by the Department of Infectious Diseases of INEN who were diagnosed with NHL between the years 2004 and 2015. Forty-seven patients were diagnosed with NHL and continued treatment at the institute during that time period. Patients who died prior to receiving ART were excluded from the study. All patients have at least one recorded CD4 count and viral load assessment close to the time of NHL diagnosis,(i.e. within 3 months prior to or 3 months after diagnosis).

Medical records were reviewed for demographic data (address, age, sex, date of birth, place of birth, nationality, marital status, occupation) and risk factors for sexually transmitted diseases. In each case, serum lactate dehydrogenase (LDH), hemoglobin (HB), aspartate aminotransferase (AST) and albumin at diagnosis were collected. Hepatitis B and C, syphilis and HTLV status as assessed on admission to the institute were also recorded.

Assessment of HIV viral load and CD4 count close to diagnosis was performed by the National Institute of Health of Peru. Pathological diagnoses were reviewed by the Department of Pathology at INEN, and lymphomas were classified according to the classification of the World Health Organization. Ki67 was classified as high in those tumors that have a percentage equal or greater than 80. The Ann Arbor system was used to stage the lymphomas based on CT imaging, and performance status was recorded according to the Zubrod or ECOG (Eastern Cooperative Oncology Group) scale. The International Prognostic Index (IPI) was calculated for each patient. It considers 5 parameters: age over 60 years; stage III or IV; high level of LDH; ECOG performance status of 2 or greater, and more than one extra-nodal location [9]. Patients were classified according to four levels of risk: low risk (0–1 points), low-intermediate risk (2 points), high-intermediate risk (3 points) and high risk (5 factors). An HIV score was obtained for each patient based on three independent risk factors: ECOG status of 2–4 or better, diagnosis before an AIDS-defining illness, and a low CD4 count (less than 100 cells/mm^3^). Patients were classified according to three risk levels: good (0 factors), intermediate (1 factor) and poor (2–3 factors) scores. Patients were divided into two groups: ART-exposed patients who had received ART before the diagnosis of systemic NHL (at least 3 months continuously before the diagnosis of NHL) – and ART–naïve patients.

### Statistical analysis

Differences between the groups were assessed using χ2-tests in cases of discrete variables or t-tests in cases of continuous variables. Median follow-up was calculated from the date of diagnosis to time of death or the date when the patient was last seen in clinic. Analyses of the effects of patient characteristics at time of diagnosis upon OS were estimated using the Kaplan-Meier method, and compared using log-rank tests. Cox proportional-hazards regression analyses were used to evaluate multivariate analyses. All factors associated with a *p*-value < 0.05 by univariate analyses and variables that were significant according to the literature were included in the multivariate analyses. *p*-values < 0.05 were considered statistically significant. All analyses were performed with.

## Results

The characteristics of the patients are summarized in Table 1. The majority of patients were ART-naive (72.34%). The groups do not differ significantly in age at diagnosis (37.5 and 40.8 years, respectively), gender (64.7 and 76.9% male, respectively) and marital status (58.8 and 61.5% single, respectively). Over three-quarters (76.9%) of ART-exposed patients diagnosed with NHL in this cohort were born in Lima, the capital city of Peru. This was a significant difference from the ART-naïve cohort, in which fewer than half of the patients were natives of Lima (*p* = 0.039).

**Table 1:** Characteristics and risk behaviors of HIV-positive patients with non-Hodgkin lymphoma

Characteristics	Art-naive	Art-exposed	Total
Cases	34 (72.3%)	13 (27.7%)	47 (100%)
NHL as AIDS-defining	31 (91.1%)	12 (92.3%)	43 (91.5%)
Homo / bisexual	10 (29.4%)	3 (23.1%)	13 (27.7%)
Age at first SI	16.3 ± 2.5	16.4 ± 2.4	16.3 ± 2.4
Number of sexual partners	16.8 ± 19.4	53.4 ± 136.8	27.4 ± 75.2
Rape victim	4 (8.5%)	0	4 (8.51%)
Prostitution	3 (8.82%)	2 (15.38%)	5 (10.64%)
SI abroad	5 (14.71%)	1 (7.69%)	6 (12.67%)
Orogenital intercourse	15 (44.12%)	6 (46.15%)	21 (44.68%)
Anal intercourse	15 (44.12%)	6 (46.15%)	21 (44.68%)
Drug addiction	5 (7.14 1%)	1 (7.69%)	6 (12.77%)
Blood transfusion^*^	2 (5.88%)	4 (30.77%)	6 (12.67%)
Previous surgery	10 (29.41%)	6 (46.15%)	16 (47.05%)
Tooth extraction	26 (76.47%)	11 (84.61%)	37 (78.72%)
Travel or residence in endemic area	12 (35.29%)	3 (23.07%)	15 (44.11%)
History of STDs	13 (38.24%)	7 (53.84%)	20 (42.55%)
Co-infections	5 (14.71%)	5 (38.46%)	10 (21.28%)
Hepatitis B	2 (5.88%)	1 (7.69%)	3 (6.39%)
Syphilis	1 (2.94%)	0	1 (2.12%)
HTLV-1	1 (2.94%)	4 (30.77%)	5 (10.64%)

^*^p = 0.025 SI:Sexual Intercourse, *STD* Sexually transmitted diseases

At the time of diagnosis, patients who were ART-exposed prior to NHL diagnosis had a significantly higher hemoglobin level (*p* = 0.01). No significant difference in NHL sub-types was noted between the two cohorts. Thirty (30) cases were diffuse large B-cell lymphoma (DLBCL); (15 were of germinal center b-cell (GCB) origin, 13 were activated B cell (ABC) and the rest did not have a determined origin). Molecular subtype was not determined on the remaining 17 tumor specimens. There was no significant difference in molecular subtype based on prior ART exposure. Likewise, no significant difference was found in Ki67, bone marrow involvement, IPI or HIV score between the two mentioned groups **(**Table 2).

**Table 2:** Characteristics at diagnosis of NHL in HIV-positive patients

Characteristics	N (hn/ph)	Art-naive	Art-exposed	Total
CD4 T cells < 100 (cells/mm^3^)	47 (34/13)	19 (55.88%)	5 (38.46%)	24 (51.06%)
HIV-RNA > 400 (copies/mL)	47 (34/13)	23 (67.65%)	7 (53.85%)	30 (63.83%)
LDH > 330 (mg /dL)	47 (34/13)	31 (91.18%)	12 (92.31%)	43 (91.49%)
Hemoglobin < 10 (g/L)	47 (34/13)	7 (20.59%)	7 (53.85%)	14 (29.79%)
WBC (× 10^9^/L; mean +/− SD)	46 (34/12)	7.09 ± 0.87	5.69 ± 0.86	6.73 ± 0.68
Lymphocytes (× 10^9^/L; mean +/− SD)	45 (34/11)	20.49 ± 2.26	25.73 ± 4.21	21.77 ± 1.99
Albumin< 3 (U/L)	47 (34/13)	8 (23.53%)	3 (23.08%)	11 (23.4%)
AST (IU/L; mean +/− SD)	47 (34/13)	70.68 ± 13.71	36.42 ± 5.64	61.11 ± 10.24
Presence of B symptoms	47 (34/13)	16 (47.06%)	5 (38.46%)	21 (44.68%)
Ki67 (%)	36 (27/9)	23 (85.18%)	9 (100%)	32 (88.88%)
Tumor stage III-IV	47 (34/13)	7 (20.59%)	6 (46.15%)	13 (27.66%)
BM Involvement	42 (32/10)	4 (12.5%)	1 (10%)	5 (10.64%)
ECOG≥ 2	47 (34/13)	11 (32.35%)	4 (30.77%)	15 (31.91%)
HIV SCORE ≥ 2	47 (34/13)	23 (67.65%)	6 (46.15%)	29 (61.07%)
IPI ≥ 3	47 (34/13)	18 (52.94%)	5 (38.46%)	23 (48.93%)
TYPE OF NHL	47 (34/13)			
DLBC		21 (61.76%)	9 (69.23%)	30 (63.83%)
Burkitts		5 (14.7%)	2 (15.38%)	7 (14.89%)
MALT		2 (5.88%)	0	2 (4.26%)
Anaplastic		6 (17.65%)	2 (15.38%)	8 (17.02%)

*HN* HAART naïve, *PH* prior HAART, *WBC* white blood cells, *BM* bone marrow, *DLBC* diffuse large B cell, *MALT* Mucosa-associated lymphoid tissue (MALT). * ***p*** **= 0.026**

The average patient follow-up was 48 months. Estimated 1-year and 5-year overall survival (OS) was 61.7 and 36.2%, respectively. The average OS of ART-naïve patients was significantly higher than that of ART-exposed patients (57.1 versus 21.1 months, respectively); (*p* = 0.0176). According to Kaplan Meier and log rank analysis, OS was significantly reduced in patients who had a serum albumin level lower than 3, advanced stage (III-IV) and previous ART treatment (*p* = 0.0183, 0.0045, 0.045 respectively).

In univariate analysis, prognostic factors for an HIV-positive patient with NHL were high IPI score, poor ECOG performance status (greater than 1), advanced tumor stage and a low level of albumin. Also, it was found that a high IPI score and poor ECOG performance status were prognostic factors in ART–naïve patients. Albumin level was a prognostic factor in ART-exposed patients. On multivariate analysis, prognostic factors for an HIV-positive patient with NHL were previous ART therapy, advanced tumor stage and low level of albumin. Age appeared to be the main prognostic factor in ART-naive patients, whereas low level of albumin was the main prognostic factor in ART-exposed patients (Table 3).

**Table 3:** Multivariate survival in HIV-positive patients with NHL

	TOTAL			ART-NAÏVE			ART-EXPOSED		
	HR	95% CI	*P*	HR	95% CI	*P*	HR	95% CI	HR
ART-EXPOSED	5.09	1.86–13.88	0.001						
Age > 60				50.71	2.55–1008.22	0.01			
Stage III-IV	5.89	1.15–30.1	0.033						
Albumin < 3	6.04	1.83–19.92	0.003				13.42	1.05–171.31	0.046

## Discussion

To the best of our knowledge, this is the first Latin American study comparing the survival of HIV-positive patients diagnosed with NHL based on their exposure to ART. The estimated 1-year survival was 61.7% for systemic NHL. This result is similar to other studies that suggest that the difference in survival between NHL patients with and without HIV infection is closing [10, 11]. Low albumin level, advanced stage and previous ART exposure were the predictive factors of overall survival in our study.

The majority (72.34%) of the patients included in the study had never received ART prior to their lymphoma treatment. This finding is in contrast to European studies in which the majority of patients are ART-exposed [12–17]. The fact that all patients who received ART before the diagnosis of NHL came from the capital could imply that people living in remote rural areas are less likely to receive ART. This highlights the inequality in the geographical distribution of health care in a developing country like Peru.

Five-year survival in HIV-infected patients diagnosed with systemic NHL was low compared to European cohort studies [12, 18], but similar to other Latin American studies [19, 20]. Although the difference in survival between NHL patients with and without HIV is generally felt to be improving, we see that the OS in developing countries like Peru continues to lag [10, 19]. This finding could be related to the fact that, unlike European countries, most Peruvian patients are diagnosed with advanced stage (63.83%), a high LDH level (91. 49%), and a high Ki67 (88.88%). The first two characteristics are part of the International Prognostic Index (IPI) and can strongly influence the low survival rate. Likewise, it should be noted that an age over 60 is another parameter of the IPI. However, in our institution, HIV-positive patients with non-Hodgkin lymphoma are young adults, and only one patient was over the age of 60 [21, 22]. In our multivariate analysis, IPI was not significant but the tumor stage was. In previous studies, the stage was the most important survival factor in these patients, and HIV - related factors did not have a significant influence on their survival [21, 23]. Furthermore, although bone marrow involvement has been considered a prognostic factor [24], in our analysis this was not statistically significant. This result could be due to the fact that few patients had bone marrow involvement.

According to our multivariate analysis, low albumin level is a prognostic factor for survival. Serum albumin has been included in the modified Glasgow score of hematological malignancies [25]. Many studies in NHL have demonstrated an association between low serum albumin level and decreased survival [26–29]. The mechanism by which low serum albumin predicts lower survival is not clear yet. It has been theorized that low serum albumin levels are associated with poor nutritional state, increased inflammatory response to the tumor, and increased cytokine release. This is the first study that finds low serum albumin level as an independent survival factor in HIV-positive patients with NHL [30].

ART–naïve patients had better OS compared with patients who had received ART prior to their diagnosis with NHL. This is consistent with a similar study by the COHERE group [12] that reported that ART-naïve patients could start ART as an effective rescue therapy. In contrast, patients who develop NHL while being on ART had a worse prognosis. Although our study confirmed a better prognosis in ART-naïve patients, it is important to note that ART decreases the overall incidence of NHL and reduces mortality in HIV patients [1].

This is the first study to evaluate the OS of HIV-positive patients with systemic NHL in Latin America and the first that found the value of serum albumin as an independent prognostic factor in the survival of these patients. However, the present study has several limitations. First, the study included only patients who survived long enough to receive treatment (ART and chemotherapy) after diagnosis and excluded patients who never received treatment. This requirement could lead to an overestimate of the OS of ART-naïve patients. Second, adherence to treatment was not evaluated. Third, the small number of patients available for this study results in a low statistical power. Despite its retrospective nature and the mentioned limitations, we consider that this study is valuable because it describes a population for which little data are currently available in the literature.

## Conclusions

We conclude that tumor stage, serum albumin and exposure to ART are prognostic factors associated with survival in HIV-positive patients with NHL. ART-naïve patients have a significantly greater OS than those who received ART prior to their diagnosis of NHL. However, evidence is clear that ART reduces the incidence of NHL in HIV-positive patients. Further evaluation of these risk factors in a prospective study in this population is warranted.

## Abbreviations

ART: Antiretroviral therapy
HIV: Human immunodeficiency virus
NHL: Non-Hodgkin’s lymphoma
